# The Anti-Inflammatory Effects of Lipoxygenase and Cyclo-Oxygenase Inhibitors in Inflammation-Induced Human Fetal Glia Cells and the Aβ Degradation Capacity of Human Fetal Astrocytes in an *Ex vivo* Assay

**DOI:** 10.3389/fnins.2017.00299

**Published:** 2017-05-30

**Authors:** Rea Pihlaja, Merja Haaparanta-Solin, Juha O. Rinne

**Affiliations:** ^1^PET Preclinical Laboratory, Turku PET Centre, University of TurkuTurku, Finland; ^2^Medicity Research Laboratory, University of TurkuTurku, Finland; ^3^Turku PET Centre, Turku University HospitalTurku, Finland

**Keywords:** Alzheimer's disease, astrocyte, microglia, neuroinflammation, arachidonic acid pathway

## Abstract

Chronic inflammation is a common phenomenon present in the background of multiple neurodegenerative diseases, including Alzheimer's disease (AD). The arachidonic acid pathway overproduces proinflammatory eicosanoids during these states and glial cells in the brain gradually lose their vital functions of protecting and supporting neurons. In this study, the role of different key enzymes of the eicosanoid pathway mediating inflammatory responses was examined *in vitro* and *ex vivo* using human fetal glial cells. Astrocytes and microglia were exposed to proinflammatory agents i.e., cytokines interleukin 1-β (IL-1β) and tumor necrosis factor (TNF-α). ELISA assays were used to examine the effects of inhibitors of key enzymes in the eicosanoid pathway. Inhibitors for 5-lipoxygenase (5-LOX) and cyclo-oxygenase 2 (COX-2) in both cell types and 5-, 12-, and 15-LOX-inhibitor in astrocytes reduced significantly IL-6 secretion, compared to exposed glial cells without inhibitors. The cytokine antibody array showed that especially treatments with 5, -12, and -15 LOX inhibitor in astrocytes, 5-LOX inhibitor in microglia and COX-2 inhibitor in both glial cell types significantly reduced the expression of multiple proinflammatory cytokines. Furthermore, human fetal astrocytes and microglia were cultured on top of AD-affected and control human brain sections for 30 h. According to the immunochemical evaluation of the level of total Aβ, astrocytes were very efficient at degrading Aβ from AD-affected brain sections *ex vivo*; simultaneously added enzyme inhibitors did not increase their Aβ degradation capabilities. Microglia were not able to reduce the level of total Aβ during the 30 h incubation time.

## Introduction

The amyloid cascade hypothesis is the most widely accepted theory to account for the appearance of Alzheimer's disease (AD); amyloid plaques are one of the main hallmarks of the disease in conjunction with the formation of neurofibrillary tangles and the development of a chronic proinflammatory condition (Selkoe and Hardy, 2016). Microglia and astrocytes are the glial cells in the brain; these cells play a crucial role in ensuring that the inflammatory responses and levels of amyloid beta (Aβ) in the central nervous system (CNS) are not excessive. However, one feature of AD related pathology is that the neurotoxic forms of Aβ start to accumulate, which when combined with the chronic proinflammatory status, reduces the Aβ degradation capacity of the glial cells, leading to massive Aβ accumulation (Koenigsknecht-Talboo and Landreth, 2005; Lee and Landreth, 2010). After decades of intensive and wide-ranging research efforts, the recent success with Aβ antibody treatments represents a much-awaited positive signal that it may be possible to devise effective drugs to prevent the accumulation of neurotoxic forms of amyloid-beta (Aβ) in AD (Selkoe and Hardy, 2016). However, several other pathogenic pathways are also potentially involved as AD pathogenesis develops further. The vicious and destructive cycles mediated by chronic inflammatory processes have proven difficult to halt, not only in AD but also in other neurodegenerative diseases.

During the acute inflammatory response, the arachidonic acid (AA) pathway produces proinflammatory eicosanoids; in many neurodegenerative diseases, this AA pathway has become chronically hyperactivated (Rao et al., 2011). In the brain, primarily monoacylglycerol lipase (MAGL) hydrolyzes the endocannabinoid 2-arachidonoylglycerol to AA, which is further metabolized into a wide range of proinflammatory eicosanoids (Nomura et al., 2011). MAGL inhibition, which not only reduces the level of eicosanoids but also simultaneously elevates those of neuroprotective endocannabinoids, has been able to decrease AD-mimicking pathology in some animal models (Chen et al., 2011; Piro et al., 2012; Pihlaja et al., 2015).

The purpose of this study was to examine the role of different key enzymes in the eicosanoid pathway in mediating the inflammatory responses *in vitro* and *ex vivo* in human fetal glial cells. As an extension to the previous research in this field, in the present study we manipulated different levels of the AA pathway to elucidate the mechanisms of inflammatory responses. We also evaluated Aβ degradation in human fetal astrocytes and microglia. In an *in vitro* assay, we exposed glial cells to proinflammatory cytokines and used ELISA and cytokine antibody arrays to reveal the properties of different eicosanoid route key enzymes in mediating the inflammatory response of the cells.

It has been shown previously that during a 18 h incubation in *in vitro* conditions, primary adult human astrocytes and microglia bind and take up more avidly synthetic oligomeric (Aβ_oligo_) than fibrillar Aβ (Aβ_fib_) (*p* < 0.001); furthermore microglia were more efficient in taking up Aβ_fib_ in comparison to astrocytes (*p* < 0.05) (Mulder et al., 2014). In our previous studies, we have shown that cultured adult mouse astrocytes are capable of degrading Aβ deposited in human AD brain sections (Pihlaja et al., 2008). In the present pilot study, we cultured fetal human astrocytes and microglia on top of AD-affected and control human brain sections in order to determine whether cultured, immature glial cells possessed the capacity to degrade Aβ in this *ex vivo* assay. Simultaneously, the key regulatory enzymes of the eicosanoid pathway which had been tested initially in the *in vitro* experiments were inhibited now in this *ex vivo* assay. The goal was to examine whether there are key enzymes, which if inhibited, would facilitate the Aβ degrading capacity of the glial cells. Furthermore, we evaluated whether Aβ degradation mechanisms by these fetal glial cells involved extracellular and/or intracellular processes.

## Materials and methods

### In vitro

Commercially available primary human fetal astrocytes isolated from cerebral cortex (Cat: SC1800 ScienceCell Research Laboratories Inc., USA) and primary human fetal microglia isolated from the total brain tissue (Cat: 3H1900, 3H Biomedical, Sweden), age 12–15 weeks, were cultured in 96-well plates with appropriate medium and supplements (5 × 10^4^/well). The cells were incubated first for 24 h at 37°C without any drug exposures in culture medium to allow the cells to attach onto the bottom of the well. The medium was removed and specific inhibitors of the AA pathway regulatory enzymes were added. These were the cyclo-oxygenase 2 (COX-2) inhibitor nimesulide 100 μM, the COX-1/COX-2-inhibitor diclofenac 10 μM, the 5-lipoxygenase (5-LOX) inhibitor zileuton 100 μM, the 5, -12 and -15 LOX inhibitor 2-TEDC 10 μM, (all from Tocris) and the MAGL inhibitor JZL184 10 μM, (AdooQ Biosciences). The drugs were added separately to the different wells (*n* = 3) with the pre-warmed cell culture medium without serum and incubated for 1 h at 37°C. Subsequently, interleukin 1-β (IL-1β, 50 ng/ml) and tumor necrosis factor (TNF-α, 50 ng/ml) were both added to the wells and incubated for 30 h at 37°C. The medium was collected, centrifuged at 2,000 g × 10 min and stored at −80°C. The cells were rinsed with PBS, fixed with 4% formalin for 20 min, rinsed and stored in PBS at +4°C.

In the experiments measuring the principal inflammatory response and the effect of inhibitors, the level of secreted interleukin-6 (IL-6) was determined from the culture medium by ELISA (Human IL-6 kit, Abcam #ab178013) according to the instructions provided by the manufacturer. In the same experiment, the remaining medium was tested with for Human Cytokine Antibody Array (Abcam #ab133997). The parallel samples from the *in vitro* test (*n* = 3) were pooled and diluted (medium samples from microglia 1:5 and from astrocytes 1:2.5) in this experiment and one treatment was undertaken with each membrane (altogether 8 membranes/kit).

In the immunochemistry, the cells were permeabilized first for 3 × 5 min with PBST (0.05% Tween-20 in PBS), the endogenous peroxidase was blocked using 0.1% BSA in PBST for 30 min. Rabbit pAb GFAP (DAKO, Agilent Technologies U.S.A.) was added at a dilution of 1:500 in 5% NGS in PBST and incubated overnight at +4°C with slow shaking. The cells were washed 3 × 5 min in PBST and secondary antibody goat anti-rabbit Alexa 568 (Abcam, UK) was added at a dilution of 1:500 in 5% NGS in PBST and they were then incubated for 2 h at RT. The cells were washed, dried briefly and mounted with VectaShield mounting medium containing DAPI (Vector Laboratories, U.S.A.). In all, 5-6 images were taken in a single session from parallel samples using Zeiss Axiovert 200M microscope with Zeiss AxioCam MRc camera and Axiovision Release 4.8.1 software. The images were quantified using per area (%) method (Image J 1.43 U, Wayne Rasband, NIH USA).

### Ex vivo

#### Human brain material

The human brain tissue samples to be used in the *ex vivo* experiments were kindly provided by Dr. Federico Roncaroli and Dr. Djordje Gveric from Multiple Sclerosis and Parkinson's Tissue Bank, Imperial College, London. The approval for the use of human tissue material was obtained from The Peer Review Panel of the Parkinson's UK Brain Bank, Imperial College, London and The Ethics Committee of Southwest Finland Hospital District. Turku PET Centre standard operation procedures #7001 and #7601 regarding handling and discarding biohazardous material were followed throughout the study.

The human post-mortem, snap-frozen brain samples had been isolated from an 85 year old male; according to his autopsy report, he had received a neuropathological diagnosis which identified high AD related neuropathological changes with isocortical and allocortical amyloid and tau pathology, CERAD C, neuritic Braak stage VI, Thal Aβ phase 4/5 and with moderate cerebral amyloid angiopathy type 2. The control samples were isolated from a non-demented 89 year old female, with the neuropathological diagnosis referring to moderate microvascular pathology and aging-related changes. The brain blocks were cut under sterile conditions from the anterior frontal cortex area along the brain surface in a cryostat (20 μM) on round glass coverslips, transferred into 48-well culture plates and stored in −20°C until used.

#### *Ex vivo* assay with inhibitors of key regulatory enzymes in the eicosanoid pathway

The sections were incubated for 1 h at RT, before seeding human astrocytes and microglia carefully added drop by drop on top of the sections at a density of 10^5^ cells/well and incubated for 24 h to allow the cells to attach on top of the sections. The culture media was removed and fresh, serum-free media with the previously mentioned inhibitors of key regulatory enzymes in the eicosanoid pathway (see Section *In vitro*) were added separately to the different wells (*n* = 3) and incubated for 30 h. Control wells included either brain sections only, glia cells only or brain sections and glia cells without inhibitors. The medium was collected, centrifuged at 2,000 g × 10 min to remove any debris and stored at −80°C. The remaining cells and tissue were rinsed gently with 1 × PBS, fixed with 4% formalin for 20 min, rinsed and stored in PBS at +4°C.

In the immunohistochemical evaluation of the specimens, the sections were treated as in Section *In vitro*. but we applied double staining's using as the primary antibodies at the following dilutions mouse anti-amyloid β1-16, 6E10; 1:600 (Covance, U.S.A.), rabbit anti-amyloid β1-16, 6E10 1:600 (Absolute Antibody, Sweden) and rabbit pAb GFAP (DAKO, Agilent Technologies U.S.A.) 1:500 and mouse mAb CD68 [KP1] 1:100 (Abcam). The secondary antibodies were (dilutions in brackets) donkey pAb anti-mouse DyLight 488, (1:600), goat anti-rabbit IgG Alexa 488 (1:500), goat anti-rabbit IgG Alexa 568 (1:500) and donkey pAb anti-Mouse Alexa 568 (1:500) (all from Abcam). The cells were washed, dried briefly and mounted with VectaShield mounting medium with DAPI (Vector Laboratories, U.S.A.). Images were taken from 3 parallel samples (*n* = 6–12 each sample for astrocytes, 5–6 for microglia) using Zeiss Axiovert 200M microscope and analyzed as described above (see Section *In vitro*).

#### *Ex vivo* array with broad spectrum protease inhibitors

Human astrocytes and microglia were cultured on top of human AD or control brain sections at a density of 7 × 10^4^ cells/well and incubated for 30 h. Glial cells without human brain sections, brain sections without glial cells or medium only were used as control samples. The medium was collected, centrifuged at 1.2 × 10^4^ rpm for 2 min and stored at −70°C. The remaining brain section and cells were washed 2 × PBS, fixed with 3.7% formalin and washed again and finally washed and stored at +4°C in PBS.

Aβ_42_ oligomers (Innovagen) were prepared beforehand according to Stine et al. (2011). Briefly, the lyophilizate was diluted into 1,1,1,3,3,3-hexafluoro-2-propanol (HFIP) to a concentration of 1 mM, evaporated overnight at RT and stored at −20°C. When used, DMSO was added to obtain a the stock concentration of 100 μM and stored at +4°C for 24 h before diluting to the desired final concentration with the medium.

Medium from the control samples described above or from AD or control brain sections incubated with glia cells were added to a 96-well plate (*n* = 2). The irreversible serine protease inhibitor 4-(2-aminoethyl)benzenesulfonyl fluoride hydrochloride (AEBSF) 1 mM, the irreversible cysteine protease inhibitor N-[N-(L-3-transcarboxyirane-2-carbonyl)-L-leucyl]-agmatine (E-64) 10 μM, the metalloprotease inhibitor ethylenediaminetetraacetic acid disodium salt solution (EDTA-Na_2_) 1 mM or the aspartyl peptidase inhibitor (3S,4S)-4-amino-3-hydroxy-6-methylheptanoic acid (Pepstatin A) 10 μM, all from Sigma-Aldrich, Germany, were added into the wells together with the medium collected previously from the wells, where AD/control sections were being incubated together with astrocytes or microglia. The wells were incubated at +37°C for 20 min before adding the Aβ_42_ peptides prepared previously.

The wells were incubated for 24 h, samples were collected quickly on ice and stored at −70°C until ELISA measurement. ELISA was undertaken according to the manufacturer's instructions (Human Aβ_42_ ELISA Kit, #KHB3441, Invitrogen).

### Statistics

When three or more groups were compared, one-way analysis of variance followed by Dunnett's multiple comparison test was used. All data represent the mean ± SD, except for the array analysis, see Section *In vitro*. Statistical analysis were performed with GraphPad Prism® version 5.01 software (GraphPad Software Inc., CA). Statistical significance was assumed if *p* < 0.05.

## Results

### Lipoxygenases and cyclo-oxygenase 2 regulate the proinflammatory response in human fetal glia

**Figure 1:**
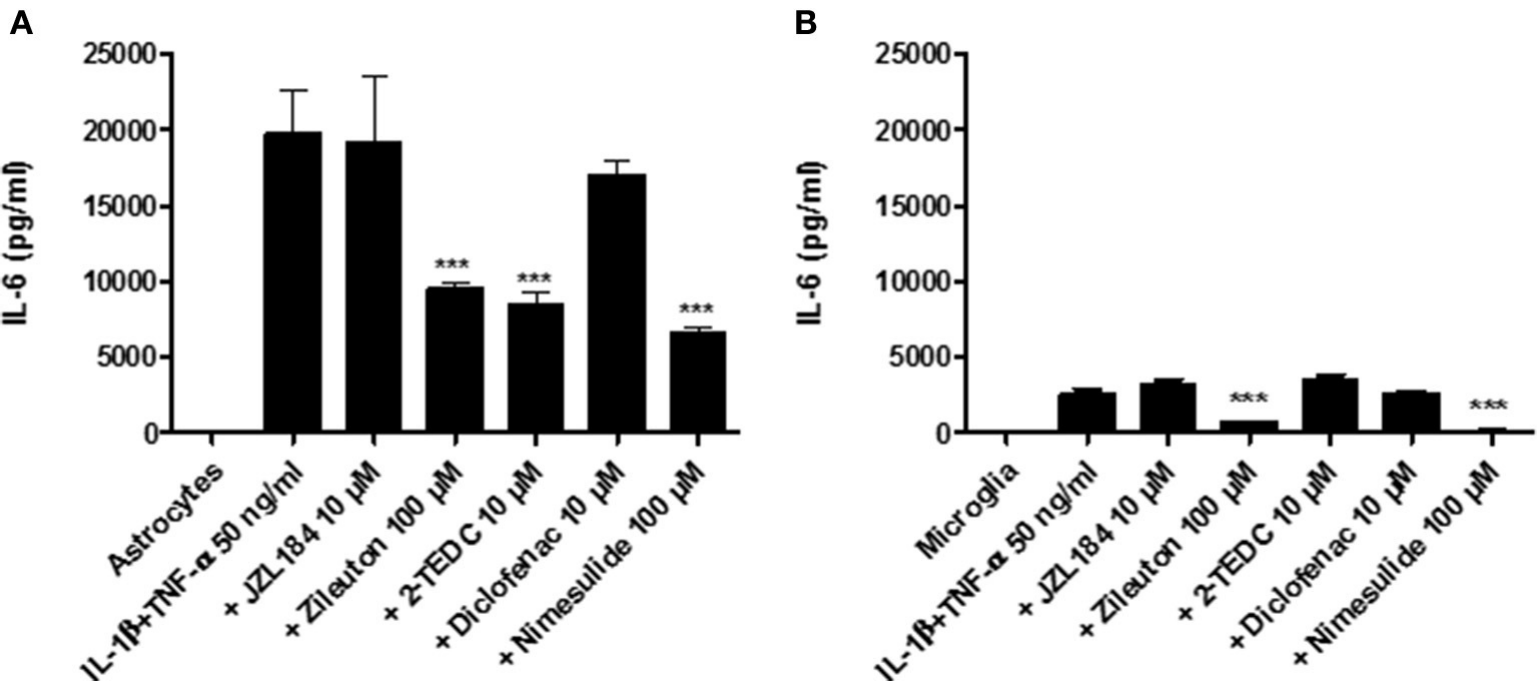
Human fetal astrocytes and microglia were induced to adopt a proinflammatory state by their simultaneous exposure to 50 ng/ml IL-1β and TNF-α and the effect of different inhibitors of key regulatory enzymes in the eicosanoid pathway leading to IL-6 secretion was measured. After 30 h incubation **(A)** the 5-LOX-inhibitor zileuton, the 5-, 12-, and 15-LOX-inhibitor 2-TEDC and the COX 2-inhibitor nimesulide added to the astrocytes and **(B)** The 5-LOX-inhibitor and the COX 2-inhibitor added to microglia reduced significantly the IL-6 secretion, when compared to glial cells not exposed to inhibitors (*p* < 0.001, *n* = 3). ***p < 0.001.

Simultaneous exposure to IL-1β (50 ng/ml) and TNF-α (50 ng/ml) for 30 h induced the highest IL-6 secretion from astrocytes (Figure 1A) and microglia (Figure 1B) with respect to the proinflammatory agents as evaluated by ELISA (see Section *In vitro*). The treatment with the 5-LOX-, 5-, 12-, and 15-LOX and COX-2 inhibitors significantly inhibited the IL-6 secretion from astrocytes (*p* < 0.001) in comparison to astrocytes not exposed to inhibitors (Figure 1A). Exposure with IL-1β and TNF-α did not induce as extensive IL-6 secretion in microglia, but also in these cells, inhibitors of 5-LOX- and COX-2 reduced IL-6 secretion significantly (*p* < 0.001), when compared to exposed microglia without inhibitors (Figure 1B).

**Figure 2:**
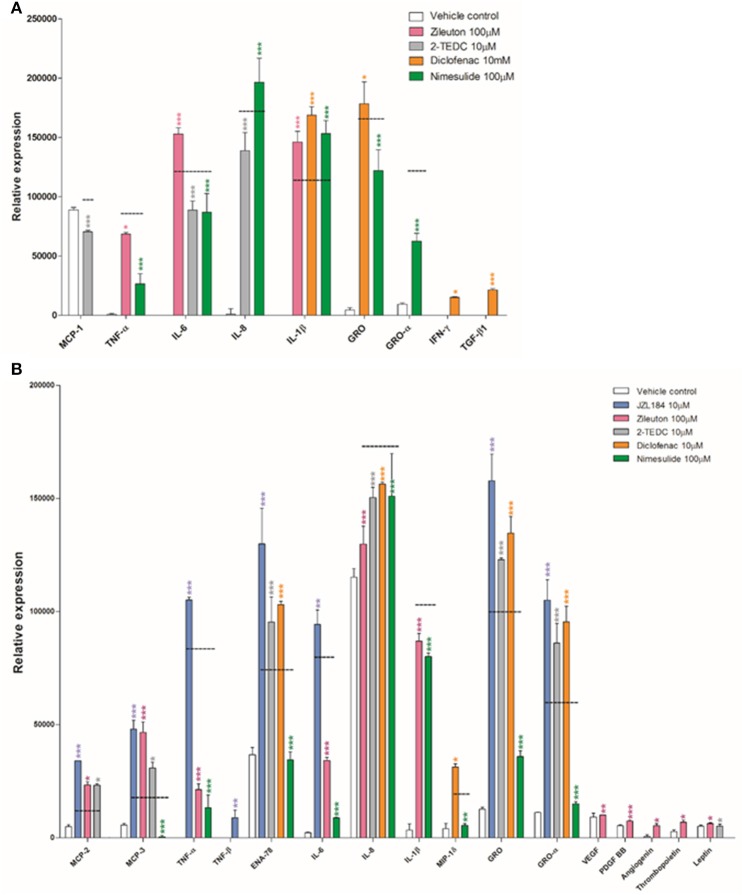
**(A)** Human fetal astrocytes exposed to TNF-α and IL-1β (50 ng/ml), were incubated with the 5, -12, and -15 LOX inhibitor 2-TEDC (gray) which reduced significantly the secretion of MCP-1, IL-6 and IL-8 (*p* < 0.001); the COX-2 inhibitor, nimesulide (green) reduced significantly the secretion of TNF-α, IL-6, GRO and GRO-α (*p* < 0.001) but at the same time increased the secretion of IL-1β and IL-8 (*p* < 0.001), when compared to astrocytes not exposed to inhibitors (black dashed line). The COX-1/COX-2-inhibitor, diclofenac (orange) increased the levels of IL-1β, TGF-β (*p* < 0.001), GRO and IFN-γ (*p* < 0.05) when compared to astrocytes not exposed to inhibitors. **(B)** In human fetal microglia exposed to TNF-α and IL-1β, the COX-2 inhibitor, nimesulide reduced significantly the secretion of MCP-3, TNF-α, ENA-78, IL-6, IL-8, IL-1β, GRO, and GRO-α (*p* < 0.001), MCP-2 and MIP-1δ (*p* < 0.01) and the 5-LOX inhibitor, zileuton (pink) reduced significantly the secretion of TNF-α, IL-6, IL-8, and IL-1β (*p* < 0.001), in comparison to microglia not exposed to inhibitors. *n* = 3. *p < 0.05, **p < 0.01, ***p < 0.001.

Some of the tested inhibitors changed significantly the secretion level of the cytokines as measured in the human cytokine array from the medium of exposed glia cells. The parallel samples (*n* = 3) from each treatment were pooled from the *in vitro* experiment (see Section *In vitro*) and added to each membrane of the array kit, i.e., the result from each membrane represents the mean value of this pool. However, in the array, there are two parallel antibody spots for each cytokine to ensure that all of the steps in the array are performed technically correctly and no unspecific reactions occur; the standard deviation describes only the deviation between these parallel spots. The array reveals the expression of 42 cytokines known to be involved in diverse pro- and anti-inflammatory states. All the raw data obtained are included in Supplementary Material Data Sheets 1–10 and Supplementary Images 1–4. Figure 2 shows only the results from those cytokines in which the signal intensity of the antibody spot (i.e., its expression) differed significantly due to enzyme inhibitor treatment, when compared to the corresponding cytokine expression level in the membrane treated with exposure only (black dashed line). Treatment with the 5, -12, and -15 LOX inhibitor reduced significantly the secretion of monocyte chemoattractant protein-1 (MCP-1), IL-6 and IL-8 (*p* < 0.001) in astrocytes, compared to astrocytes not exposed to inhibitors (Figure 2A). Similarly, the addition of the COX-2 inhibitor reduced significantly the secretion of inflammatory cytokines TNF-α, IL-6 and the chemokines, growth regulated protein (GRO) and GRO-α (*p* < 0.001) but at the same time, it increased the secretion proinflammatory cytokine IL-1β and anti-inflammatory cytokine IL-8 (*p* < 0.001) in exposed astrocytes. Exposure to the COX-1/COX-2-inhibitor tended to increase the level of proinflammatory cytokines and growth factors, especially IL-1β, tumor growth factor-β (TGF-β; *p* < 0.001), GRO and interferon-γ (IFN-γ; *p* < 0.05) in comparison to exposed astrocytes with no inhibitors.

Exposure to the COX-2 inhibitor also reduced very significantly the secretion of monocyte chemoattractant protein-3 (MCP-3), TNF-α, epithelial neutrophil activating peptide (ENA-78), IL-6, IL-8, IL-1β, GRO, and GRO-α (*p* < 0.001) but also MCP-2 and macrophage inflammatory protein-1δ (MIP-1δ) (*p* < 0.01) in exposed microglia, when compared to microglia not exposed to inhibitors (Figure 2B). In addition in microglia, the 5-LOX inhibitor significantly reduced the secretion of TNF-α, IL-6, IL-8, and IL-1β (*p* < 0.001).

### Human fetal astrocytes degrade Aβ *ex vivo*

**Figure 3:**
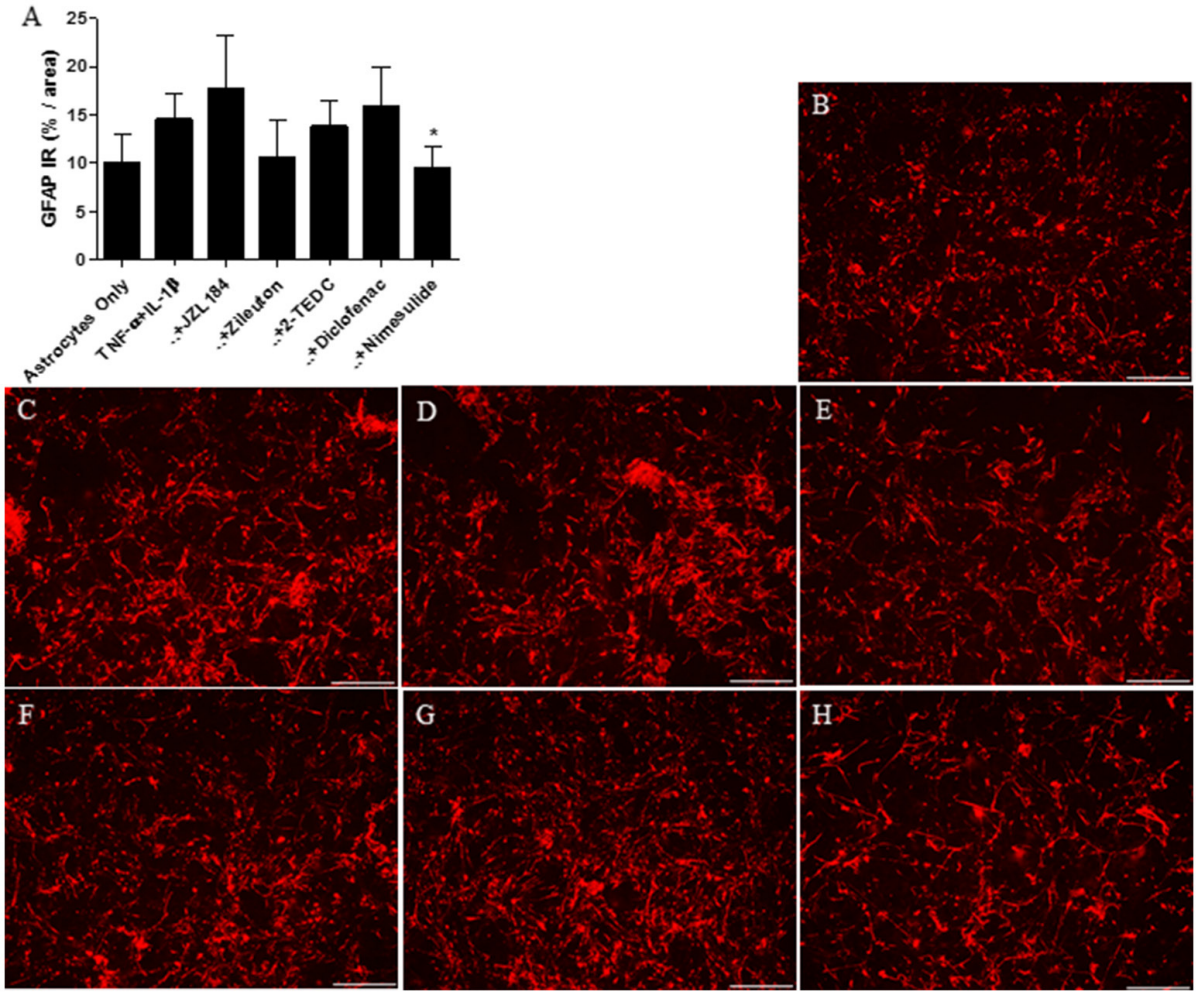
**(A)** After 30 h incubation, treatment with the COX 2-inhibitor nimesulide (*p* < 0.05, *n* = 4–6) reduced significantly the expression of GFAP in IL-1β and TNF-α (50 ng/ml) exposed human fetal astrocytes, in comparison to astrocytes not exposed to the inhibitors. Representative images indicating the level of GFAP immunoreactivity (red) in astrocytes incubated with **(B)** medium only, **(C)** with TNF-α and IL-1β added into the medium or these exposures together with **(D)** MAGL-inhibitor JZL184, **(E)** 5-LOX inhibitor zileuton, **(F)** 5, -12, and -15 LOX inhibitor 2-TEDC, **(G)** COX-1/COX-2-inhibitor diclofenac or **(H)** COX-2 inhibitor nimesulide. Scale bars: 40 μM. *p < 0.05.

In the *in vitro* experiment, the level of astrocyte specific GFAP was measured from the cells after 30 h incubation. Treatment with the COX 2-inhibitor reduced significantly (*p* < 0.05) the GFAP immunoreactivity in astrocytes exposed to IL-1β and TNF-α (50 ng/ml) when compared to exposed astrocytes with no inhibitors (Figure 3A). In addition, treatment with the 5-LOX-inhibitor tended to reduce GFAP expression but the effect was not statistically significant. Figures 3B–H show the level of GFAP immunoreactivity in each of the treatments.

**Figure 4:**
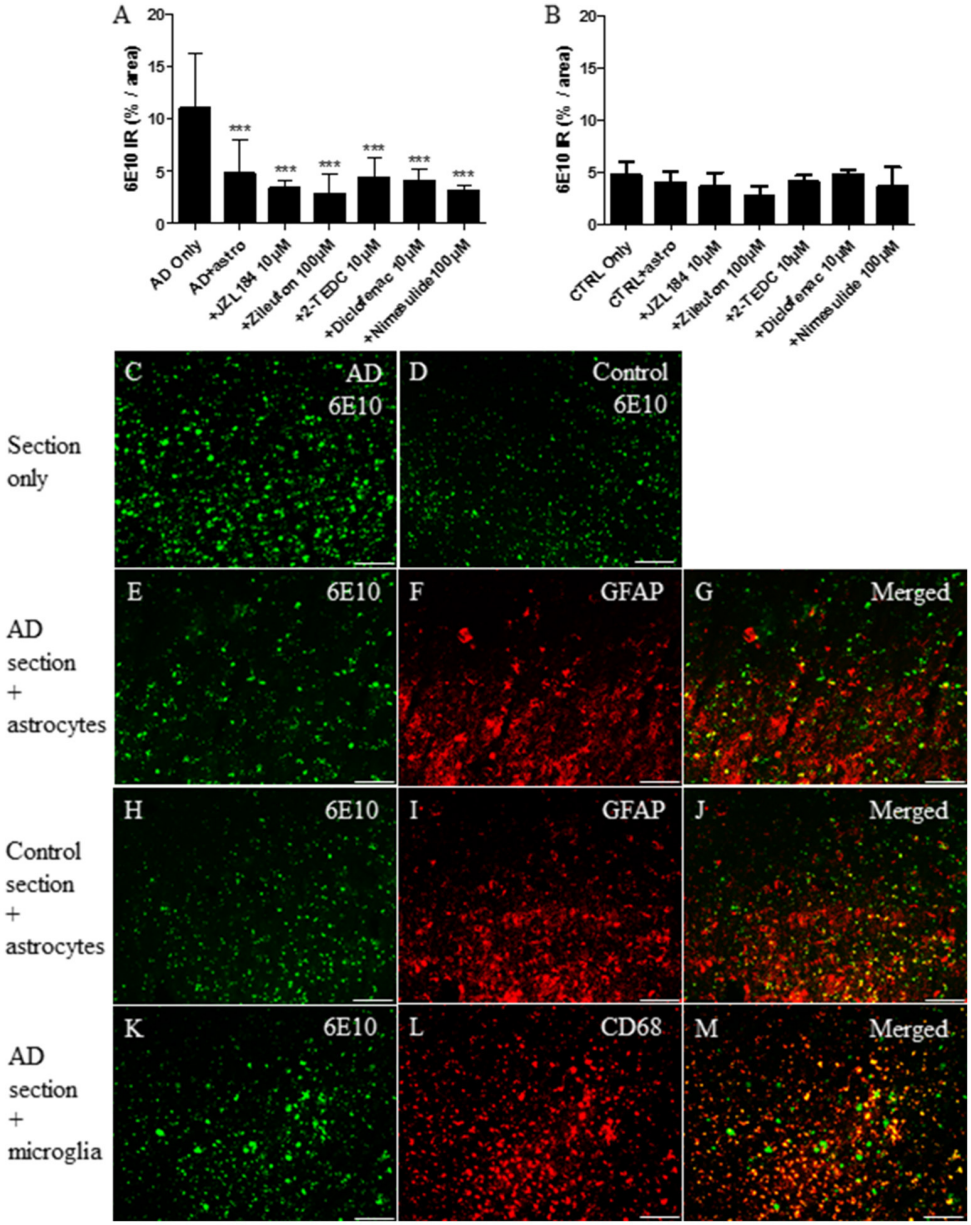
After 30 h incubation, (F) GFAP immunoreactive cultured human fetal astrocytes (red) reduced significantly (A,E) total Aβ burden (as assessed via 6E10 immunoreactivity, green) from human AD brain sections, when compared to (A,C) sections incubated with medium only (*p* < 0.001, *n* = 6–12). **(A)** However, treatment with inhibitors of the regulatory enzymes in the eicosanoid pathway did not increase further Aβ degradation, when compared to astrocytes incubated on top of the AD sections without inhibitors. Astrocytes cultured on top of the AD-affected brain section became arranged typically in groups near to the Aβ deposits **(E–G)**. Neither astrocytes nor simultaneously added inhibitors had any effect on the typically smaller level of total Aβ in control brain sections incubated under similar conditions **(B,D,H–J)**. Also, **(L)** CD68 immunoreactive fetal human microglia (red) attached on top of the **(K)** human AD brain sections, but did not reduce the level of total Aβ burden, with or without inhibitors (data not shown). Panels **(G,J,M)** represent merged images. Scale bars: 40 μM. ***p < 0.001.

Considering *ex vivo* assay, the level of 6E10 immunoreactivity was measured from human AD and control brain sections after 30 h incubation with astrocytes or microglia with or without exposure to the inhibitors of regulatory enzymes in the eicosanoid pathway. The results showed that cultured astrocytes significantly reduced the level of 6E10 which reflected the total Aβ burden (*p* < 0.001) in AD affected brain sections when compared to sections with no astrocytes (Figures 4A,C,E). The level of 6E10 immunoreactivity in control sections alone remained low and neither astrocytes nor inhibitors had any effect on the level of total Aβ (Figures 4B,D,H–J). GFAP immunoreactive astrocytes (Figure 4F) cultured on top of the AD-affected brain section arranged typically in groups near to the Aβ deposits (Figure 4G). In our study, CD68 immunoreactive microglia (Figure 4L) cultured on top of AD (Figures 4K,M) or control brain sections did not change the total Aβ burden, with or without inhibition of the key regulatory enzymes in the eicosanoid pathway (data not shown).

### Human fetal astrocytes degrade Aβ intracellularly

**Figure 5:**
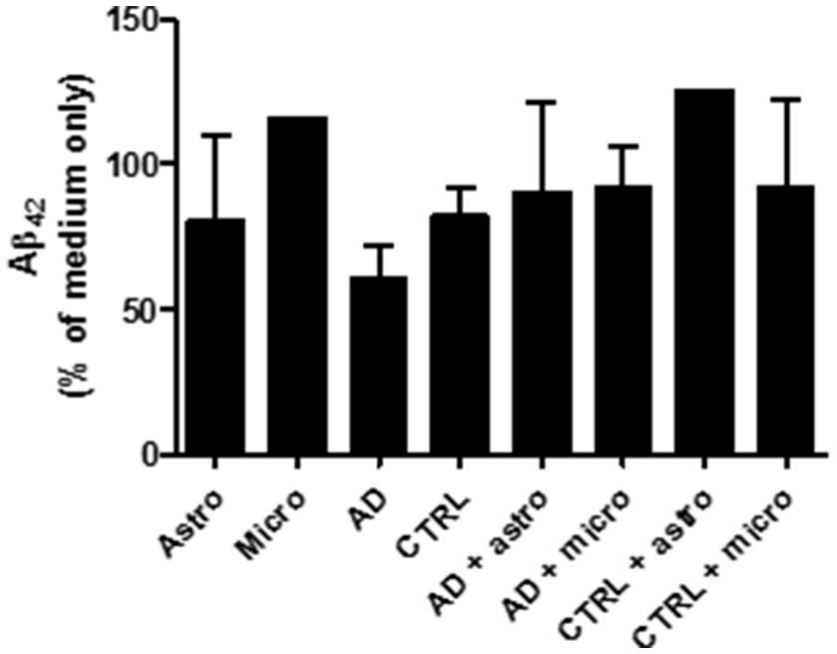
The culture medium was collected from different *ex vivo* conditions presented in the diagram and incubated with oligomeric Aβ_42_. After 24 h incubation, the culture medium did not notably reduce the level of Aβ_42_. Thus, the cultured glial cells incubated on top of the human AD sections did not secrete notable levels of Aβ_42_ degrading proteases outside the cell, suggesting that the Aβ degradation was principally an intracellular process. In addition, in the samples where only astrocytes were cultured with brain sections without protease inhibitors, there was no increase in the level of added Aβ_42_ peptides when these were compared to medium only collected from the top of the brain sections (with no astrocytes). This indicates that the astrocytes had degraded intracellularly the Aβ derived from the sections and did not simply displace it into the medium.

According to the results obtained from the human Aβ_42_ ELISA assay (see Section *Ex vivo* array with broad spectrum protease inhibitors), collected medium from different culture conditions (glia cells only, brain sections only or brain sections with cultured glia cells) did not reduce the level of oligomeric Aβ_42_ added later into the collected medium samples (Figure 5). Also, the broad spectrum inhibitors of the different protease families did not affect for Aβ_42_ levels which became released later into the cell culture medium. Thus, in our *ex vivo* experiment, the cultured glial cells incubated on top of the human AD sections did not secrete notable levels of Aβ_42_ degrading proteases outside the cell, suggesting that the Aβ degradation was principally an intracellular process. In addition, in the samples where only astrocytes were cultured with brain sections without protease inhibitors, there was no increase in the level of added Aβ_42_ peptides when these were compared to medium only collected from the top of the brain sections (with no astrocytes). This indicates that the astrocytes had degraded intracellularly the Aβ derived from the sections and did not simply displace it into the medium. However, these preliminary results require further studies.

## Discussion

Many neurodegenerative diseases, including AD are associated with a chronic inflammatory environment in the CNS. In healthy brain, astrocytes and microglia perform many important functions including the regulation of inflammatory responses and the elimination of excess toxic Aβ levels. According to the inflammation-centered view, persistent overproduction of inflammatory cytokines during the early stages of AD pathology and even before the accumulation of excess levels of Aβ, impairs the normal function of the glial cells. One of the many negative and detrimental effects is the impairment of the crucial Aβ degradation capacity of these cells, e.g., by impairing the function of receptors phagocytosing Aβ (Hickman et al., 2008; Mawuenyega et al., 2010; Heneka et al., 2015). Furthermore, there is a reduction and down-regulation in the activities of Aβ degrading proteases neprilysin (NEP), insulin degrading enzyme (IDE) and angiotensin converting enzyme (ACE) already in the early stages of AD, partly due to reactive oxygen species although this decline in enzyme activity also occurs during normal aging (Savaskan et al., 2001; El-Amouri et al., 2008).

As far as we are aware, this is the first study investigating the contributions of different key enzymes in the eicosanoid pathway to the expression of a vast array of cytokines in inflammation-induced fetal human glia cells. We also performed a small-scale pilot study to test how these cells would react when cultured on top of Aβ-burdened human brain tissue isolated from either AD-affected or non-demented control cases. For example, it is not known how rapidly a massive but short-term Aβ exposure during glial phagocytosis induces negative effects on the Aβ degradation capacity of glial cells. In this assay, we also included inhibitors of the key enzymes in the eicosanoid pathway, to observe whether only short-term inhibition of certain enzymes would exert any further effect on Aβ degradation.

In our experiment, simultaneous exposure with IL-1β and TNF-α produced a dramatic and rapid proinflammatory response in both astrocytes and microglia; this represented the ”basal level” to which the anti-inflammatory effect of the different enzyme inhibitors was individually compared.

In microglia, the COX-2 inhibitor evoked the most efficient anti-inflammatory effect, notably reducing the level of all the measured proinflammatory cytokines. The 5-LOX inhibitor also reduced significantly the level of many common proinflammatory cytokines, e.g., TNFα, IL-6, IL-8, and IL-1β. Thus, COX-2 and 5-LOX inhibitors seem to possess the greatest potential anti-inflammatory effect in human fetal microglia. This confirms the importance of these enzymes in regulating proinflammatory responses in certain inflammatory conditions, including some neurodegenerative diseases (Phillis et al., 2006; Hwang et al., 2013; Palumbo and Bosetti, 2013; Kong et al., 2016). Based on our results, it seems that these enzymes exert similar beneficial anti-inflammatory effects during the early stage of glial cell development. However, COX-2 is also involved in endocannabinoid metabolism (metabolizing both donoylethanolamine (AEA) and 2-AG) as well as generating multiple active prostaglandin analogs possessing both proinflammatory and anti-inflammatory effects. Although COX-2 inhibition impacts on many crucial pathways, the importance of this finding with respect to specific neurodegenerative diseases remains largely unknown (Alhouayek and Muccioli, 2014).

The MAGL inhibitor induced rather unexpected results, increasing further the expression of several exposure–induced proinflammatory cytokines, when compared to untreated microglia. However, it has been shown that MAGL expression disappears from cultures microglia after LPS treatment (Kallendrusch et al., 2012) suggesting that this cytokine-inducing effect was not due to MAGL inhibition but possibly a compound specific effect. In addition, the 5, -12, and -15 LOX and COX1/COX-2 inhibitors increased further the proinflammatory effect by inducing the expression of C-X-C-motif family chemokines GRO, GRO-α (CXCL1) and ENA-78 (CXCL5, epithelial-derived neutrophil-activating peptide 78). These chemokines are important messengers involved in maintaining the physiology of the nervous system (for example, involvement in migration and proliferation of the cells) but they also rapidly activate a vast array of immune cells in times of neuroinflammation (Liu et al., 2014). In contrast, in our previous *in vitro* test using LPS-, IFN-γ-, and Aβ_42_-exposed adult mouse astrocytes and microglia, transient entire eicosanoid pathway inhibition with JZL184 generally reduced the proinflammatory responses of the glial cells, although only the levels of NO, IL-1β, Iba1, and GFAP were measured (Pihlaja et al., 2015). That study did not measure the drug's effect on the expression of so many cytokine, but it nonetheless indicated that there may be profound differences between mouse and human glial cells in their inflammatory responses, at least in *in vitro* conditions. This difference between our studies is also consistent with many other observations from the field (Smith and Dragunow, 2014), highlighting the importance of using human-derived cells when developing anti-inflammation therapies.

With regard to astrocytes exposed *in vitro*, the results from the cytokine array were rather conflicting. The inhibitor of 5, -12, and -15 LOX displayed the most clear anti-inflammatory effect by reducing significantly the expression of IL-6 and IL-8. The treatment with the COX-2 inhibitor also impaired significantly the expression of most of the measured proinflammatory cytokines, but surprisingly, simultaneously evoked a further substantial increase in the levels of two cytokines, IL-8 and IL-1β.

With respect to IL-8, it has been shown previously that simultaneous exposure to IL-1 and TNF-α strongly increases the expression of IL-8 in human astrocytes and treatment with a 5-LOX inhibitor could reduce this effect (Qazi et al., 2011). Indeed, in our experiment in glial cells IL-1β and TNF-α-induced expression of IL-8 was highest in comparison with the other cytokines. Furthermore, treatment with almost all inhibitors reduced significantly its expression in microglia. However, in astrocytes only, treatment with the 5, -12, and -15 LOX inhibitor reduced significantly the level of IL-8, whereas the COX-2 inhibitor increased its expression significantly. IL-8 is a very efficient chemotactic and proinflammatory cytokine which affects all migratory immune cells, especially activating neutrophil granulocytes as well as exerting a wide array of proinflammatory functions (Qazi et al., 2011). However, it possesses also some anti-inflammatory properties e.g., it can inhibit adhesion between leucocytes and activated endothelial cells (Nourshargh et al., 1992).

Previously, it has been shown in an *in vitro* assay that human adult astrocytes prefer to degrade oligomeric Aβ over its fibrillar forms, although they can degrade both Aβ forms (Nielsen et al., 2010). Neprilysin (NEP) and insulin-degrading enzyme (IDE) are the most common Aβ degrading proteases in glial cells; in addition SCARB1 has been shown to be one of the receptors responsible for binding and taking up Aβ (Mulder et al., 2012).

In our study, human astrocytes isolated from fetal cortex were able to degrade Aβ efficiently in brain specimens from AD affected patients during a relatively short incubation time. In our previous *ex vivo* studies using mouse astrocytes, only adult astrocytes exhibited any ability to degrade Aβ from brain sections isolated from AD affected patients, in contrast neonatal astrocytes showed no such response (Pihlaja et al., 2008). We speculate that human fetal astrocytes exploit the same mechanisms in binding and degrading Aβ as their mature counterparts, although this proposal remains to be confirmed. However, our results suggest that the fetal astrocytes might have matured relatively quickly, considering their rapid responses for tissue-derived, toxic Aβ. However, the addition of drugs inhibiting key enzymes in the eicosanoid pathway did not further increase the Aβ degrading properties of astrocytes, at least during the short incubation time of 30 h.

Consistent with multiple previous studies observing poor Aβ degradation capacity of microglia possibly due to low hydrolytic activity in their lysosomes (Frackowiak et al., 1992; Majumdar et al., 2007), in our *ex vivo* assay we observed that these cultured cells were not able to reduce the level of total Aβ from the sections, when compared to sections incubated with medium only. The addition of macrophage colony-stimulating factor (M-CSF) usually increases the Aβ-degrading capacity of microglia by acidifying the cellular environment and thus increasing the hydrolytic properties of lysosomal enzymes (Majumdar et al., 2007; Mulder et al., 2014). M-CSF is typically added into the culture medium of microglia, but in our study it did not have any effect on Aβ degradation. Furthermore, the inhibitors of the key enzyme of the eicosanoid pathway did not induce Aβ degradation of microglia, consistent with the results obtained with the astrocytes.

In both *in vitro* and *ex vivo* experiments, the incubation time used was relatively short and the maturation stage of the fetal glia may have a profound effect on the overall results, especially when attempting to compare the present results with experiments conducted with adult glial cells (harvested from either healthy or AD-affected donors) with prolonged incubation time. In addition, genomic variations between glial cells isolated from different individuals may be responsible for differences in their inflammatory responses.

According to previous studies from the field and our own results, the key regulatory enzymes in the eicosanoid pathway i.e., COX-2, 5-LOX and 5-, 12-, and 15-LOX, appear to have an important role in mediating the proinflammatory responses already during an early stage of human development, providing further evidence that in the future it may be possible to manipulate proinflammatory pathways for therapeutic purposes.

## Author contributions

RP designed the experiments, performed and analyzed the experiments, coordinated collaborations, and wrote the manuscript. MH and JR analyzed the data and coordinated collaborations. All authors checked and approved the final manuscript.

### Conflict of interest statement

The authors declare that the research was conducted in the absence of any commercial or financial relationships that could be construed as a potential conflict of interest.

## Supplementary material

Supplementary Image 1 Human fetal astrocytes exposed to TNF-α and IL-1β (50 ng/ml), were incubated with the 5, −12, and −15 LOX inhibitor 2-TEDC (gray) which reduced significantly the secretion of MCP-1, IL-6, and IL-8 (*p* < 0.001); the COX-2 inhibitor, nimesulide (green) reduced significantly the secretion of TNF-α, IL-6, GRO, and GRO-α (*p* < 0.001) but at the same time increased the secretion of IL-1β and IL-8 (*p* < 0.001), when compared to astrocytes not exposed to inhibitors (black column with black dashed line). The COX-1/COX-2-inhibitor, diclofenac (orange) increased the levels of IL-1β, TGF-β (*p* < 0.001), GRO, and IFN-γ (*p* < 0.05) when compared to astrocytes not exposed to inhibitors. ^*^*p* < 0.05, ^**^*p* < 0.01, ^***^*p* < 0.001.

Supplementary Image 2 Identical graph to Supplementary Image 1, but the black column has been replaced by black dashed line to make the data easier to follow.

Supplementary Image 3 In human fetal microglia exposed to TNF-α and IL-1β (50 ng/ml), the COX-2 inhibitor, nimesulide reduced significantly the secretion of MCP-3, TNF-α, ENA-78, IL-6, IL-8, IL-1β, GRO, and GRO-α (*p* < 0.001), MCP-2 and MIP-1δ (*p* < 0.01), and the 5-LOX inhibitor, zileuton (pink) reduced significantly the secretion of TNF-α, IL-6, IL-8, and IL-1β (*p* < 0.001), in comparison to microglia not exposed to inhibitors (black column with black dashed line) *n* = 3. ^*^*p* < 0.05, ^**^*p* < 0.01, ^***^*p* < 0.001.

Supplementary Image 4 Identical graph to Supplementary Image 1, but the black column has been replaced by black dashed line to make the data easier to follow.

## Abbreviations

2-TEDC: 2-(1-Thienyl)ethyl 3,4-dihydroxybenzylidenecyanoacetate
AA: arachidonic acid
AD: Alzheimer's disease
Aβ: amyloid beta
Aβ_fib_: fibrillar amyloid beta
Aβ_olig_: oligomeric amyloid beta
BSA: bovine serum albumin
CBR: cannabinoid receptor
CERAD: Consortium to Establish a Registry for Alzheimer's Disease
CNS: central nervous system
COX: cyclo-oxygenase
DMSO: dimethyl sulphoxide
ELISA: enzyme-linked immunosorbent assay
ENA: epithelial-derived neutrophil-activating peptide
GFAP: glial fibrillary acidic protein
GRO: growth regulated protein
IL: interleukin
LOX: lipoxygenase
mAb: monoclonal antibody
MAGL: monoacylglycerol lipase
MCP: monocyte chemoattractant protein
M-CSF: macrophage colony stimulating factor
MIP: macrophage inflammatory protein
NGS: normal goat serum
pAb: polyclonal antibody
PBS: phosphate buffered saline
RT: room temperature
SCARA: class A macrophage scavenger receptor
TGF: tumor growth factor
TNF: tumor necrosis factor-alpha.

## References

[B1] Alhouayek M. Muccioli G. G. (2014). COX-2-derived endocannabinoid metabolites as novel inflammatory mediators. Trends Pharmacol. Sci. 35, 284–292. 10.1016/j.tips.2014.03.001 24684963

[B2] Chen X. Zhang J. Chen C. (2011). Endocannabinoid 2-arachidonoylglycerol protects neurons against β-amyloid insults. Neuroscience 178, 159–168. 10.1016/j.neuroscience.2011.01.024 21256197 PMC3052737

[B3] El-Amouri S. S. Zhu H. Yu J. Marr R. Verma I. M. Kindy M. S. (2008). Neprilysin: an enzyme candidate to slow the progression of Alzheimer's disease. Am. J. Pathol. 172, 1342–1354. 10.2353/ajpath.2008.070620 18403590 PMC2329843

[B4] Frackowiak J. Wisniewski H. M. Wegiel J. Merz G. S. Iqbal K. Wang K. C. (1992). Ultrastructure of the microglia that phagocytose amyloid and the microglia that produce beta-amyloid fibrils. Acta Neuropathol. 84, 225–233. 1414275 10.1007/BF00227813

[B5] Heneka M. T. Carson M. J. El Khoury J. Landreth G. E. Brosseron F. Feinstein D. L.. (2015). Neuroinflammation in Alzheimer's disease. Lancet Neurol. 14, 388–405. 10.1016/S1474-4422(15)70016-5 25792098 PMC5909703

[B6] Hickman S. E. Allison E. K. El Khoury J. (2008). Microglial dysfunction and defective beta-amyloid clearance pathways in aging Alzheimer's disease mice. J. Neurosci. 28, 8354–8360. 10.1523/JNEUROSCI.0616-08.2008 18701698 PMC2597474

[B7] Hwang S. H. Wecksler A. T. Wagner K. Hammock B. D. (2013). Rationally designed multitarget agents against inflammation and pain. Curr. Med. Chem. 20, 1783–1799. 10.2174/0929867311320130013 23410172 PMC4113248

[B8] Kallendrusch S. Hobusch C. Ehrlich A. Nowicki M. Ziebell S. Bechmann I.. (2012). Intrinsic up-regulation of 2-AG favors an area specific neuronal survival in different in vitro models of neuronal damage. PLoS ONE 7:e51208. 10.1371/journal.pone.0051208 23284665 PMC3527460

[B9] Koenigsknecht-Talboo J. Landreth G. E. (2005). Microglial phagocytosis induced by fibrillar beta-amyloid and IgGs are differentially regulated by proinflammatory cytokines. J. Neurosci. 25, 8240–8249. 10.1523/JNEUROSCI.1808-05.2005 16148231 PMC6725530

[B10] Kong W. Hooper K. M. Ganea D. (2016). The natural dual cyclooxygenase and 5-lipoxygenase inhibitor flavocoxid is protective in EAE through effects on Th1/Th17 differentiation and macrophage/microglia activation. Brain Behav. Immun. 53, 59–71. 10.1016/j.bbi.2015.11.002 26541818

[B11] Lee C. Y. Landreth G. E. (2010). The role of microglia in amyloid clearance from the AD brain. J. Neural Transm. 117, 949–960. 10.1007/s00702-010-0433-4 20552234 PMC3653296

[B12] Liu C. Cui G. Zhu M. Kang X. Guo H. (2014). Neuroinflammation in Alzheimer's disease: chemokines produced by astrocytes and chemokine receptors. Int. J. Clin. Exp. Pathol. 7, 8342–8355. eCollection 2014. Review. 25674199 PMC4314046

[B13] Majumdar A. Cruz D. Asamoah N. Buxbaum A. Sohar I. Lobel P.. (2007). Activation of microglia acidifies lysosomes and leads to degradation of *Alzheimer amyloid* fibrils. Mol. Biol. Cell 18, 1490–1496. 10.1091/mbc.E06-10-0975 17314396 PMC1838985

[B14] Mawuenyega K. G. Sigurdson W. Ovod V. Munsell L. Kasten T. Morris J. C.. (2010). Decreased clearance of CNS beta-amyloid in Alzheimer's disease. Science 330:1774. 10.1126/science.1197623 21148344 PMC3073454

[B15] Mulder S. D. Nielsen H. M. Blankenstein M. A. Eikelenboom P. Veerhuis R. (2014). Apolipoproteins E and J interfere with amyloid-beta uptake by primary human astrocytes and microglia *in vitro*. Glia 62, 493–503. 10.1002/glia.22619 24446231

[B16] Mulder S. D. Veerhuis R. Blankenstein M. A. Nielsen H. M. (2012). The effect of amyloid associated proteins on the expression of genes involved in amyloid-β clearance by adult human astrocytes. Exp. Neurol. 233, 373–379. 10.1016/j.expneurol.2011.11.001 22101005

[B17] Nielsen H. M. Mulder S. D. Beliën J. A. Musters R. J. Eikelenboom P. Veerhuis R. (2010). Astrocytic Ab1-42 uptake is determined by A beta-aggregation state and the presence of amyloid-associated proteins. Glia 58, 1235–1246. 10.1002/glia.21004 20544859

[B18] Nomura D. K. Morrison B. E. Blankman J. L. Long J. Z. Kinsey S. G. Marcondes M. C.. (2011). Endocannabinoid hydrolysis generates brain prostaglandins that promote neuroinflammation. Science 334, 809–813. 10.1126/science.1209200 22021672 PMC3249428

[B19] Nourshargh S. Perkins J. A. Showell H. J. Matsushima K. Williams T. J. Collins P. D. (1992). A comparative study of the neutrophil stimulatory activity *in vitro* and pro-inflammatory properties *in vivo* of 72 amino acid and 77 amino acid IL-8. J. Immunol. 148, 106–111. 1727857

[B20] Palumbo S. Bosetti F. (2013). Alterations of brain eicosanoid synthetic pathway in multiple sclerosis and in animal models of demyelination: role of cyclooxygenase-2. Prostaglandins Leukot. Essent. Fatty Acids 89, 273–278. 10.1016/j.plefa.2013.08.008 24095587

[B21] Phillis J. W. Horrocks L. A. Farooqui A. A. (2006). Cyclooxygenases, lipoxygenases, and epoxygenases in CNS: their role and involvement in neurological disorders. Brain Res. Rev. 52, 201–243. 10.1016/j.brainresrev.2006.02.002 16647138

[B22] Pihlaja R. Koistinaho J. Malm T. Sikkilä H. Vainio S. Koistinaho M. (2008). Transplanted astrocytes internalize deposited beta-amyloid peptides in a transgenic mouse model of Alzheimer's disease. Glia 56, 154–163. 10.1002/glia.20599 18004725

[B23] Pihlaja R. Takkinen J. Eskola O. Vasara J. López-Picón F. R. Haaparanta-Solin M.. (2015). Monoacylglycerol lipase inhibitor JZL184 reduces neuroinflammatory response in APdE9 mice and in adult mouse glial cells. J. Neuroinflammation 12:81. 10.1186/s12974-015-0305-9 25927213 PMC4416350

[B24] Piro J. R. Benjamin D. I. Duerr J. M. Pi Y. Gonzales C. Wood K. M.. (2012). A dysregulated endocannabinoid-eicosanoid network supports pathogenesis in a mouse model of Alzheimer's disease. Cell Rep. 1, 617–623. 10.1016/j.celrep.2012.05.001 22813736 PMC3715876

[B25] Qazi B. S. Tang K. Qazi A. (2011). Recent advances in underlying pathologies provide insight into interleukin-8 expression-mediated inflammation and angiogenesis. Int. J. Inflam, 2011:908468. 10.4061/2011/908468 22235381 PMC3253461

[B26] Rao J. S. Rapoport S. I. Kim H. W. (2011). Altered neuroinflammatory, arachidonic acid cascade and synaptic markers in postmortem Alzheimer's disease brain. Transl. Psychiatry 1:e31. 10.1038/tp.2011.27 22832605 PMC3309508

[B27] Savaskan E. Hock C. Olivieri G. Bruttel S. Rosenberg C. Hulette C.. (2001). Cortical alterations of angiotensin converting enzyme, angiotensin II and AT1 receptor in Alzheimer's dementia. Neurobiol. Aging 22, 541–546. 10.1016/S0197-4580(00)00259-1 11445253

[B28] Selkoe D. J. Hardy J. (2016). The amyloid hypothesis of Alzheimer's disease at 25 years. EMBO Mol. Med. 8, 595–608. 10.15252/emmm.201606210 27025652 PMC4888851

[B29] Smith A. M. Dragunow M. (2014). The human side of microglia. Trends Neurosci. 37, 125–135. 10.1016/j.tins.2013.12.001 24388427

[B30] Stine W. B. Jungbauer L. Yu C. LaDu M. J. (2011). Preparing synthetic Aβ in different aggregation states. Methods Mol. Biol. 670, 13–32. 10.1007/978-1-60761-744-0_2 20967580 PMC3752843

