# Supplementary figures and images for: The Anti-Inflammatory Effects of Lipoxygenase and Cyclo-Oxygenase Inhibitors in Inflammation-Induced Human Fetal Glia Cells and the Aβ Degradation Capacity of Human Fetal Astrocytes in an Ex vivo Assay
Source: Front Neurosci. 2017 May 30;11:299. doi: 10.3389/fnins.2017.00299 (PMC5447716; doi:10.3389/fnins.2017.00299)

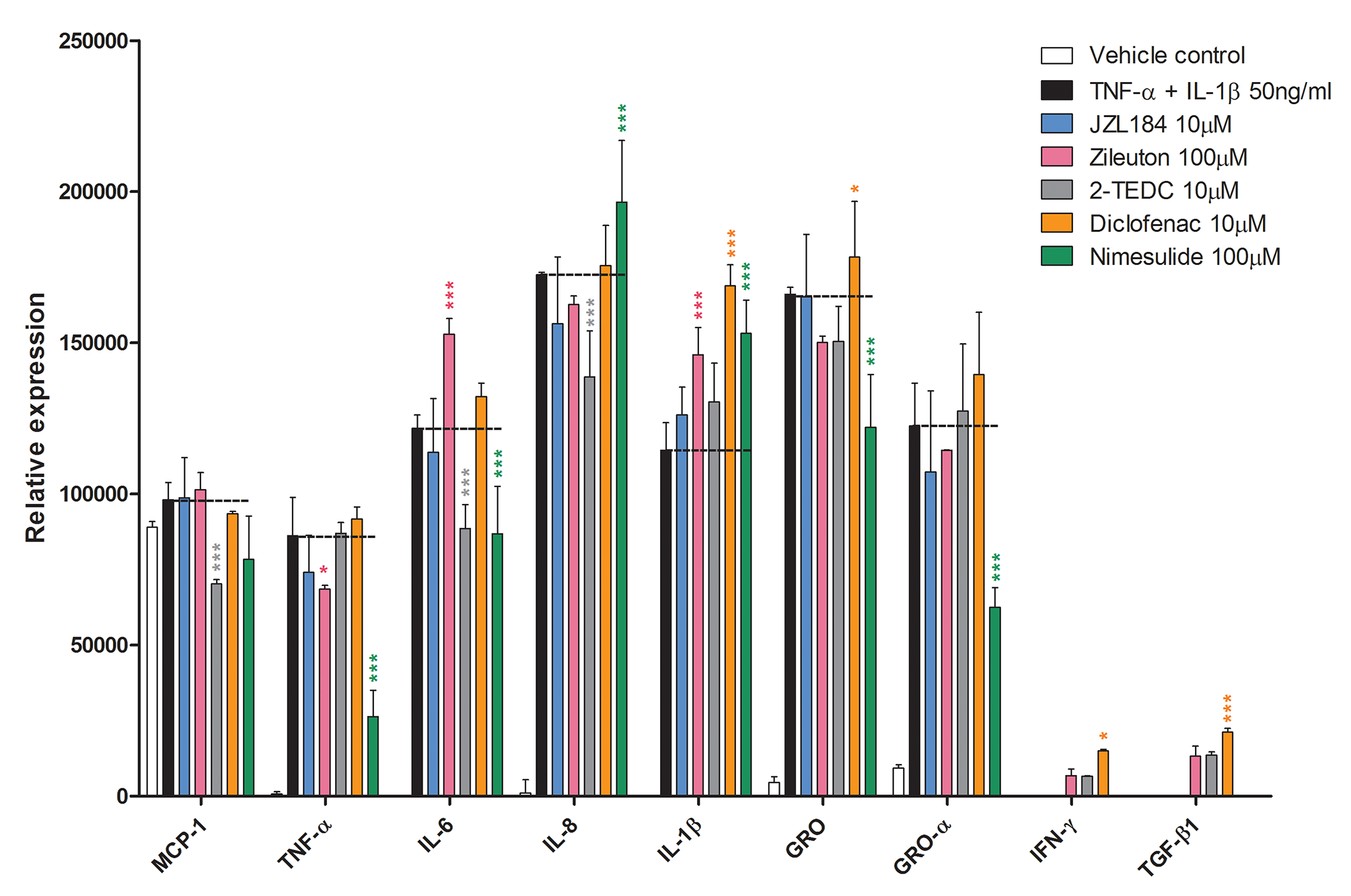

Supplement: Supplementary Image 1 — Human fetal astrocytes exposed to TNF-α and IL-1β (50 ng/ml), were incubated with the 5, −12, and −15 LOX inhibitor 2-TEDC (gray) which reduced significantly the secretion of MCP-1, IL-6, and IL-8 (p < 0.001); the COX-2 inhibitor, nimesulide (green) reduced significantly the secretion of TNF-α, IL-6, GRO, and GRO-α (p < 0.001) but at the same time increased the secretion of IL-1β and IL-8 (p < 0.001), when compared to astrocytes not exposed to inhibitors (black column with black dashed line). The COX-1/COX-2-inhibitor, diclofenac (orange) increased the levels of IL-1β, TGF-β (p < 0.001), GRO, and IFN-γ (p < 0.05) when compared to astrocytes not exposed to inhibitors. *p < 0.05, **p < 0.01, ***p < 0.001. [file Image1.tif]

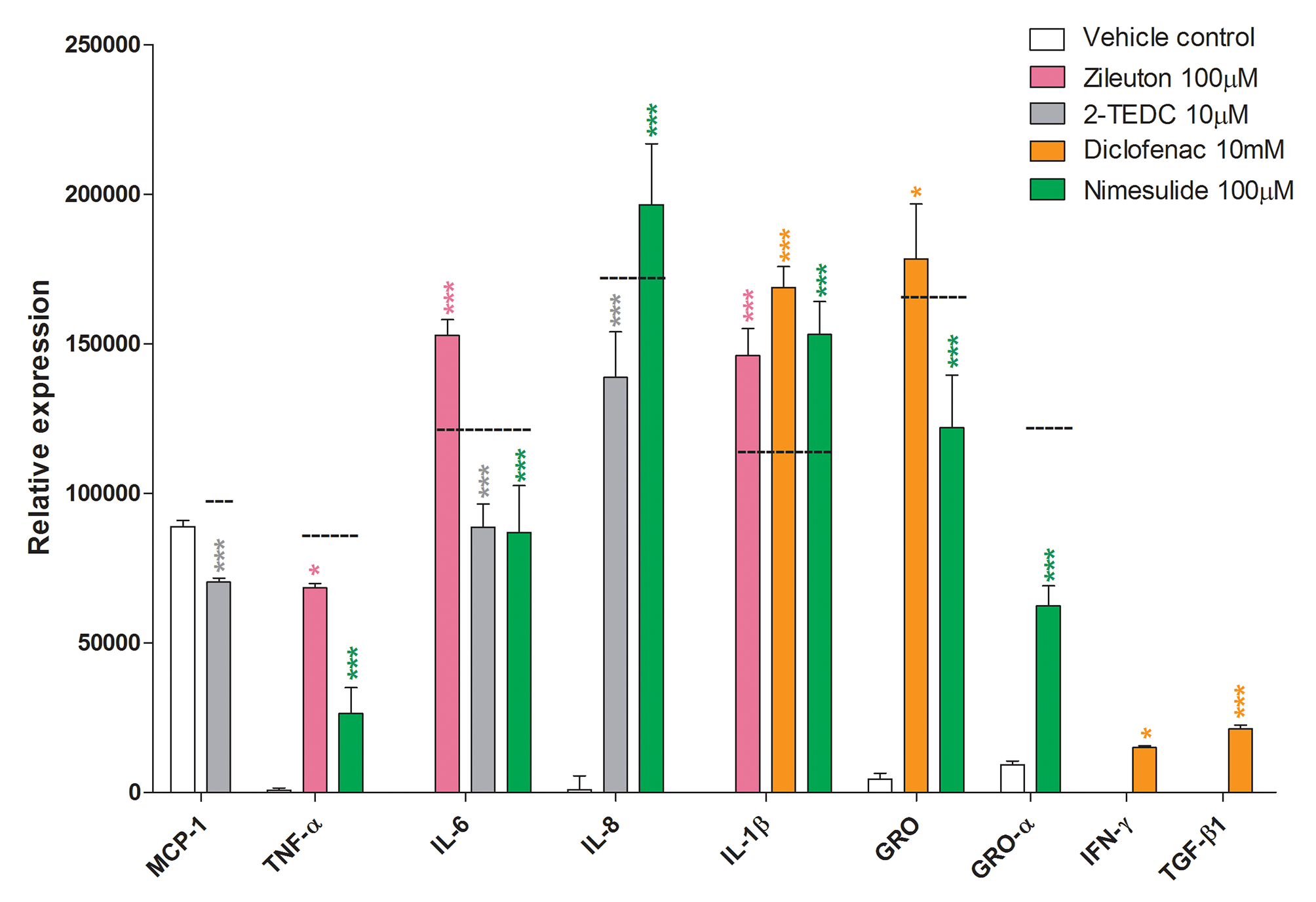

Supplement: Supplementary Image 2 — Identical graph to Supplementary Image 1, but the black column has been replaced by black dashed line to make the data easier to follow. [file Image2.tif]

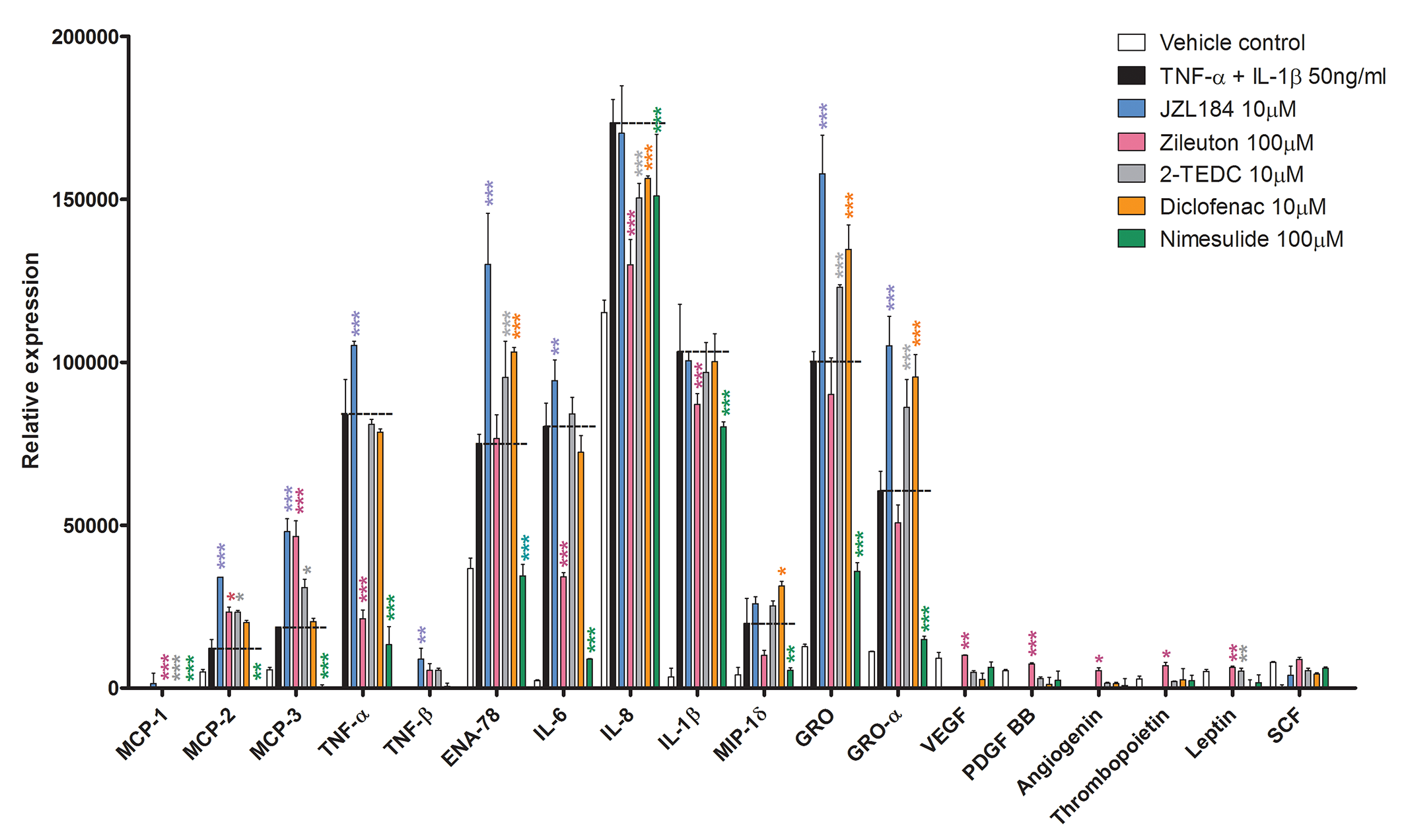

Supplement: Supplementary Image 3 — In human fetal microglia exposed to TNF-α and IL-1β (50 ng/ml), the COX-2 inhibitor, nimesulide reduced significantly the secretion of MCP-3, TNF-α, ENA-78, IL-6, IL-8, IL-1β, GRO, and GRO-α (p < 0.001), MCP-2 and MIP-1δ (p < 0.01), and the 5-LOX inhibitor, zileuton (pink) reduced significantly the secretion of TNF-α, IL-6, IL-8, and IL-1β (p < 0.001), in comparison to microglia not exposed to inhibitors (black column with black dashed line) n = 3. *p < 0.05, **p < 0.01, ***p < 0.001. [file Image3.tif]

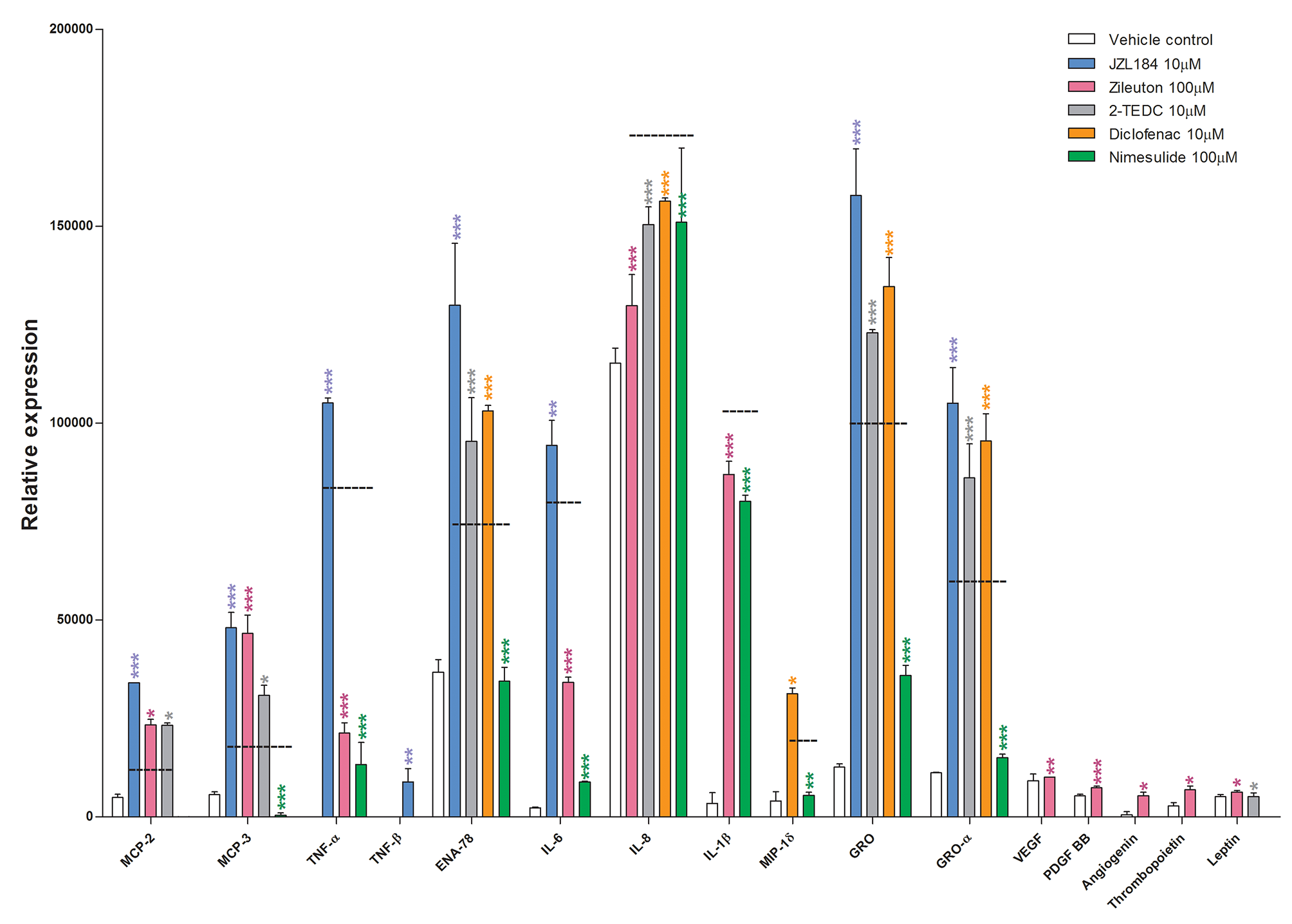

Supplement: Supplementary Image 4 — Identical graph to Supplementary Image 1, but the black column has been replaced by black dashed line to make the data easier to follow. [file Image4.tif]
